# Value of the Run-In Period to Evaluate the Safety of Conventional Trypanocidal Treatment: A Subanalysis of a Colombian Randomized Clinical Trial

**DOI:** 10.4269/ajtmh.25-0198

**Published:** 2026-04-21

**Authors:** Juan Carlos Villar, Helena Arango, Luis David Sáenz-Pérez, Antonia Camacho

**Affiliations:** ^1^ Facultad de Ciencias de la Salud, Universidad Autónoma de Bucaramanga, Bucaramanga, Colombia;; ^2^ Subdirección de Investigaciones, Fundación Cardioinfantil – Instituto de Cardiología, Bogotá, Colombia

## Abstract

When testing poorly tolerated or long-term treatments, including a run-in phase enhances clinical trial efficiency and internal validity by selecting more adherent participants. This report describes the adherence and tolerance of Colombian participants in EQUITY (a randomized, concealed, parallel-group, placebo-controlled trial testing nifurtimox and benznidazole among *Trypanosoma cruzi*–seropositive adults without cardiomyopathy). Our design included a 10-day, single-blind, placebo run-in phase. On completion, willing participants reporting good adherence (≥80%) and tolerance were randomized to any five 120-day, blinded treatments: four with either active medication, each given as 120-day half dose or 60-day full dose (followed/preceded by a randomly allocated 60-day placebo treatment), or a 120-day placebo. Side effects were compared after the run-in (day 0) between excluded and enrolled participants, and between those randomized to active medications or placebo 20 days after starting each treatment period (days 20/80). Those excluded (44/351, 12.5%) more often reported gastrointestinal (15.9/4.6%), nonspecific (13.7/4.2%), and musculoskeletal symptoms (9.1/1.6%) than those randomized. Participants given nifurtimox (*n* = 84) or benznidazole (*n* = 86) versus placebo (*n* = 126) on day 20 reported more nonspecific (15.5/10.5/4.8%, respectively) or cutaneous side effects (6.0/12.8/3.2%). When starting active treatments (transition OFF–ON, *n* = 171 and *n* = 61 in first and second 60-day treatment periods), more participants reported emerging-worsening than receding-ending side effects (49/13 and 15/5, respectively). Despite inducing a nocebo effect, the run-in phase highlighted more closely related side effects, strengthening causal inference and informing adherence and tolerance to conventional trypanocides.

## INTRODUCTION

Infection with *Trypanosoma cruzi*, endemic to the Americas, leads to approximately 12,000 deaths annually from complications that occur during its chronic phase.^1^ Each year, around 30,000 new cases are reported.^2^ The disease’s main complications typically emerge 20–30 years after the initial infection, primarily leading to a dilated cardiomyopathy (up to 30% of the infected)^3^^,^^4^ and gastrointestinal “megas” (reported in 10% in some endemic countries).^5^ The medical costs associated with managing these complications, along with loss of labor productivity and premature mortality, make Chagas disease a significant public health problem.^6^ Due to its high disease burden, limited treatment options, and the low investment in clinical research, it is classified as a neglected tropical disease.^7^ Currently, trypanocidal therapy for chronic infection is limited to two agents used as nifurtimox and benznidazole. Although either drug is recommended,^8^ supporting evidence for their use in this scenario is still weak. Moreover, limited tolerance to these agents narrows their efficacy/safety ratio, adding complexity to this treatment choice.^9^

Although several placebo-controlled trials demonstrate antiparasitic efficacy of benznidazole in children or adults, mostly from Southern Cone endemic countries,^10^^–^^13^ such data are scarce for nifurtimox. In addition, the clinical benefit of trypanocidal therapy is less supported, depending on observational studies, mostly with benznidazole, with inconsistent results across countries.^14^ In fact, the two phase-3, benznidazole placebo-controlled trials in seropositive adults conducted to date (TRAENA, with Argentinian and Bolivian participants mostly with no organ damage,^15^ and BENEFIT, with participants with established cardiomyopathy from multiple countries)^16^ failed to show clinical effect, despite confirming its antiparasitic activity. This later effect, however, in BENEFIT was seen for southern (Brazil, Argentina, and Bolivia) but not northern endemic countries (Colombia or El Salvador).

Other studies across endemic countries^17^^,^^18^ showing geographical variations in trypanocidal efficacy have also been reported, with findings that may be related to the well-known variety of parasite genotypes in the region.^19^ Despite these caveats, the importance of the disease and the proven antiparasitic effect make conventional trypanocidal therapy strongly recommended for acute or early chronic infection^20^ and especially to prevent vertical transmission in girls and women of childbearing age.^21^

Using conventional trypanocidal therapy also raises safety concerns. Up to 70% of treated patients could experience any adverse effects, and 10–27% are classified as severe.^22^^,^^23^ Intolerance to these agents often leads to treatment discontinuation (10–20% of the cases), reducing treatment effectiveness.^24^ The most commonly reported effects include cutaneous reactions (with benznidazole) and gastrointestinal disturbances and weight loss (with nifurtimox). Less frequently, benznidazole has been associated with peripheral polyneuropathy, fever, lymphadenopathy, myalgia/arthralgia, and neutropenia/thrombocytopenia.^25^^,^^26^

Among different needs, the field requires better information on the efficacy/safety ratio of trypanocidal therapy, especially for the less-studied drug (nifurtimox) and populations (those from northern endemic countries). However, low tolerance and adherence not only affect treatment administration but also the conduct of trials of trypanocidal therapy. On the other hand, open-label and observational studies may overestimate noncompliance.^27^^,^^28^ A run-in phase in clinical trials is a design tool whereby excluding the less adherent individuals before randomization enhances the study efficiency and internal validity. It has been used for less-tolerated or long-term treatments with potential adherence issues^29^^–^^31^ and often involves placebo administration, so those reporting intolerance under these conditions do not participate.^29^^–^^31^ Adding this tool improves safety assessments in trials by helping to distinguish “true” treatment-related effects,^32^ and that may be useful for the case for trypanocidal therapy.

EQUITY is a randomized trial testing the trypanocidal efficacy and safety of nifurtimox in comparison with benznidazole among *T. cruzi*–seropositive for adults from Colombia and Argentina without cardiomyopathy. The Colombian component was designed as a placebo-controlled trial, also including a 10-day, single-blind, placebo run-in phase.^33^ We report here the tolerance and adherence of its participants from Colombia while executing this protocol.

## MATERIALS AND METHODS

### Study design.

**Figure 1.**
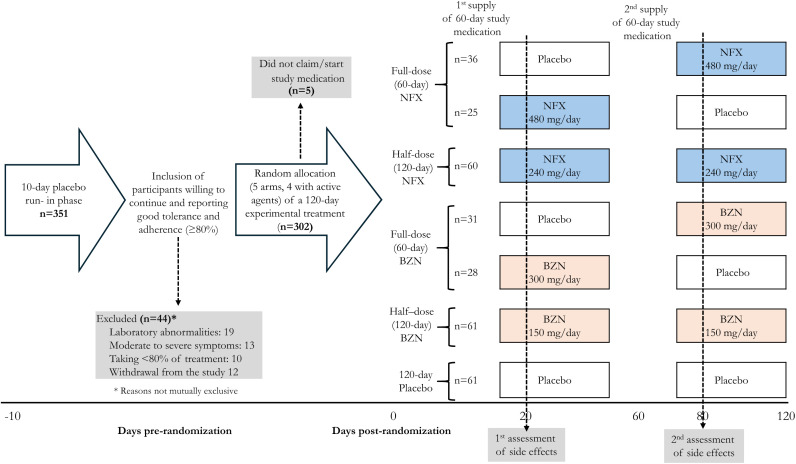
Study design describing the study timeline and distribution of the experimental treatment. BZN = benznidazole; NFX = nifurtimox.

This substudy of EQUITY is a randomized, concealed, parallel-group trial, designed to evaluate the safety and tolerance of conventional (60-day, full dose) and alternative (120-day, half dose) regimens of nifurtimox, in comparison with similar regimes of benznidazole and placebo in a blinded manner. Participants were assigned in equal proportions (1:1:1:1:1) to five groups: two nifurtimox groups (60 or 120 days), two benznidazole groups (60 or 120 days), and one placebo group (120 days). All study arms received 120-day indistinguishable treatments, consisting of two capsules administered twice daily (BID), divided into two consecutive 60-day periods. Thus, the two groups allocated to 60-day, full-dose treatments were randomly crossed-over to receive a 60-day placebo treatment to complete 120 days on study medications. The placebo run-in phase and 60-day treatment changes generated treatment transitions ON or OFF active treatment during the protocol, as shown in Figure 1.

### Inclusion and exclusion criteria.

Our eligible participants were adults 20–65 years of age who had 1) a positive serological diagnosis based on at least two positive tests using different methods (i.e., ELISA, indirect hemagglutination, or indirect immunofluorescence) conducted in the past 10 years, 2) minimal risk of reinfection (permanent residence in urban or rural areas with no history of infestation or vector transmission), and 3) the capacity to attend regular follow-up visits at study centers.

The exclusion criteria included 1) having prior treatment with benznidazole or nifurtimox, 2) participation in trials involving trypanocidal drugs, 3) showing symptoms suggesting Chagas or any cardiomyopathy or uncontrolled cardiovascular risk factors, 4) having persistent abnormalities in the kidney or liver function tests (alanine aminotransferase [ALT] levels exceeding twice the normal value or creatinine levels above 1.2 mg/dL on at least two occasions), 5) being a woman of childbearing age with a positive pregnancy test or one who refused to use effective contraceptive methods, and 6) having any accompanying health issues that, based on medical assessment, could interfere with participation, well-being, or treatment.

### Procedures.

*T. cruzi*–seropositive individuals attended a selection visit to confirm their eligibility, offer participation, and receive information about reported benefits or adverse events associated with trypanocidal therapy. Consenting individuals then entered a 10-day run-in phase, receiving two indistinguishable placebo capsules BID (mirroring the treatment scheme in the experimental groups). Participants, but not investigators or study personnel, were blind to the content of this run-in study medication. At the end of this phase, the research team evaluated treatment adherence, severity of emerging symptoms, and laboratory parameters (creatinine, ALT, and blood count). Those meeting our inclusion criteria (reiteration of their consent, having more than 80% adherence, no moderate/severe symptoms [to the level of deciding not to continue taking study medication], and no significant laboratory abnormalities) were randomly assigned to one of the five experimental groups. Study treatments were accordingly supplied and administered over two consecutive 60-day periods (Figure 1).

### Evaluation of safety and adherence.

Safety and adherence were assessed 1) at the end of the run-in phase, and 2) 20 days after each treatment period (days 20 and 80 after randomization). Laboratory abnormalities were also recorded at the end of the run-in phase and 30 days after randomization (the second follow-up visit). Our primary (safety) outcome was the incidence of a composite of moderate/severe side effects during treatment, assessed by an independent committee, based on the primary report of study physicians, both blinded for treatment allocation. The committee consisted of internal medicine physicians and general practitioners familiar with trypanocidal therapy-related side effects (it also included a dermatologist for reported cutaneous adverse reactions).

The classification was based on the Common Terminology Criteria for Adverse Events (CTCAE), endorsed by the US National Institutes of Health, which considers the subjective degree of discomfort, limitations for daily activities, or further decline of health. This tool grades different types of adverse drug reactions into a five-level ordinal scale of increasing severity (1 for mild to 5 for fatal events).^34^ Those classified as Grade 1 (mild) required no specific interventions. For those classified as Grade 2 (moderate) or Grade 3 (severe), the study drug may have been reduced or stopped (temporarily or permanently), additional medications may have been prescribed, or both. We did not have events requiring hospitalization or urgent/emergent care (some Grade 3 or higher), where participants would have been referred to the closest emergency department.

### Changes in side effects reported before and after receiving trypanocidal therapy.

The study design facilitated the evaluation of different treatment periods based on the introduction or interruption of active treatment (ON-OFF) when stopping/continuing with placebo during various protocol phases (run-in, 0–60 days, and 60–120 days). Thus, four before-and-after treatment transitions were generated within the protocol, as shown in Figure 1:

1. OFF-ON: Participants who received placebo and then began active treatment.
2. OFF-OFF: Participants who received placebo in two consecutive periods.
3. ON-ON: Participants who received active treatment in two consecutive periods.
4. ON-OFF: Participants who received active treatment followed by a period of placebo.

**Figure 2.**
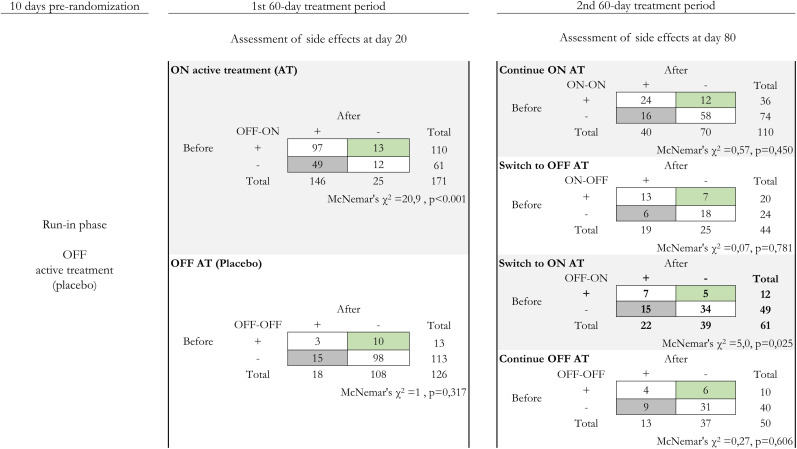
Side effects report across treatment transitions, 20 days after the start of each treatment period. Counts of participants are shown in 2-by-2, before-and-after tables representing paired observations by treatment allocation (light gray when receiving active treatment, or white when receiving placebo) that generated the transitions during the study periods (ON-OFF active treatment). The dark gray cells in the tables represent those who reported emergent/worsening side effects, whereas the counts in green represent when they were receding/ending. Counts are based on a composite outcome including symptoms of moderate-to-severe intensity, requiring a prescription of symptomatic treatments, dose adjustments, or stopping the study medication.

The contrasts generated by the distribution of participants in different experimental groups having these treatment transitions are summarized in Figure 2.

For each transition period, we recorded before–after changes in our composite safety outcome between days 0–20 and 60–80 post randomization. We explored the relationship between the incidence of symptoms and these treatment transitions throughout the study by building 2-by-2 tables with counts of participants reporting appearing (absent before–present after) or worsening versus stopping (present before–absent after) or receding side effects, as shown in Figure 2.

## STATISTICAL ANALYSES

Descriptive statistics were used to summarize the clinical and sociodemographic characteristics of participants. Continuous variables were reported as medians and interquartile ranges (IQRs), and categorical variables were presented as absolute frequencies and percentages. According to our hypothesis and assumptions for efficacy (not included in this analysis), the planned sample size for the Colombian component of EQUITY was at least 300 (about 60 participants per study arm). Our aim for this substudy was approached with three sources of comparison: 1) between initial study participants who were excluded after the run-in phase and those proceeding to randomization, 2) between participants assigned to any form of trypanocidal therapy and those receiving placebo at day 20, and 3) before–after comparisons (paired observations) within groups of participants having ON-OFF treatment transitions.

Statistical hypotheses were tested at a two-sided alpha level of 5%. Comparisons of independent groups were made with the Wilcoxon test for continuous variables and with χ^2^ for categorical variables (e.g., adherence or symptoms presentation according to the groups) or Fisher’s exact test (when expected values were <5). Within-subject, before–after changes over treatment transitions were tested with McNemar’s test for paired observations.

## RESULTS

The run-in phase began with 351 eligible individuals. Of these, 44 (12.5%; 95% CI: 9.3–16.4%) were excluded due to one or more of the following criteria: laboratory abnormalities (*n* = 19), moderate-to-severe symptoms (*n* = 13), treatment adherence below 80% (*n* = 10), or withdrawal from the study (*n* = 12). A total of 307 participants were randomized: 61 in the control group receiving placebo and 246 in the four trypanocidal therapy groups. Among the latter, five participants did not initiate medication (Figure 1).

Table 1 presents demographic and clinical characteristics of the participants. Excluded participants from the run-in phase and those randomized were not statistically different in the assessed variables. A little more than half of the participants were males, and their median age was 51 years. Participants were all urban residents, the majority (70.7%) living in low socioeconomic strata neighborhoods, and 94.6% had past rural dwelling. From the clinical perspective, numerically more of the excluded participants reported comorbidities (68.2% versus 52.4% in the randomized population, *P* = 0.072) and used chronic medication (47.7% versus 43.3%, *P* = 0.698). However, the use of medications suggesting cardiac disease (beta-blockers or diuretics) was very low, no patient reported use of antiarrhythmic drugs, and only one randomized participant had brain natriuretic peptide (BNP) levels over 100 ng/dL (median levels were 12.5 [IQR: 11.5] for the excluded and 10 [IQR: 5.5] for the randomized), reflecting that the study population was essentially free of clinically important heart disease.

**Table 1:** Demographic (above) and clinical (below) characteristics of the participants after the run-in phase

Characteristic	Total (*N* = 351)	Excluded after Run-In (*n* = 44)	Randomized (*n* = 307)	*P*-Value
Male sex, *n* (%)	200 (56.9)	24 (54.5)	176 (57.3)	0.852
Age, median (Q1–Q3)	51.0 (46.0–56.5)	49.5 (43.8–55.2)	52.0 (47.0–57.0)	0.165
Low socioeconomic status, *n* (%)	248 (70.7)	31 (70.5)	217 (70.7)	1.000
Primary or no formal education, *n* (%)	177 (50.4)	25 (56.8)	152 (49.5)	0.456
Unemployed or domestic caregiver, *n* (%)	66 (18.8)	10 (22.7)	56 (18.2)	0.612
Subsidized health regimen/no coverage, *n* (%)	105 (29.9)	15 (34.1)	90 (29.3)	0.637
History of rural housing, *n* (%)	332 (94.6)	40 (90.9)	292 (95.1)	0.276
History of any comorbidity, *n* (%)	191 (54.4)	30 (68.2)	161 (52.4)	0.072
Chronic medication use, *n* (%)	154 (43.9)	21 (47.7)	133 (43.3)	0.698
Anti-arrythmic drugs, *n* (%)	0 (0)	0 (0)	0 (0)	—
Beta blockers, *n* (%)	21 (6.0)	3 (6.8)	18 (5.9)	0.802
Diuretics, *n* (%)	12 (3.4)	2 (4.6)	10 (3.3)	0.660
Rhythm/conductive abnormalities, *n* (%)	82 (23.4)	12 (27.3)	70 (22.8)	0.512
BNP levels ≥100 pg/mL	1 (0)	0 (0)	1 (0)	0.889

BNP = brain natriuretic peptide; IQR = interquartile range.

**Figure 3.**
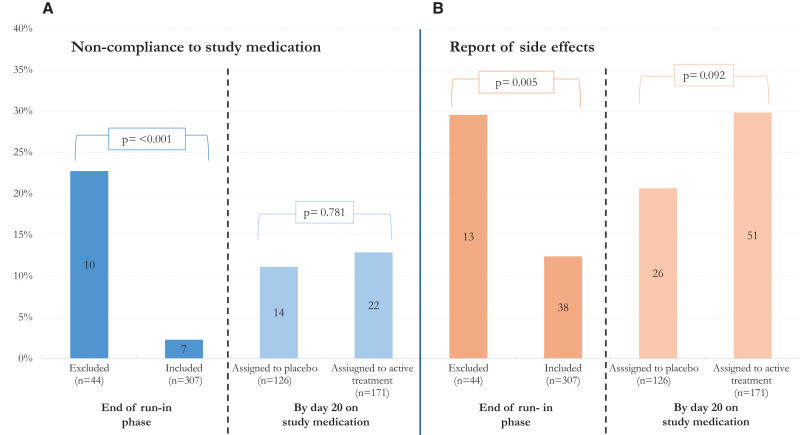
Counts of participants found with noncompliance to study medication (adherence to prescribed treatment below 80%) to the left of the figure (**A**) or reporting side effects to the right (**B**). The bars show the counts at the end of the run-in phase (left in each panel) if excluded or included in the trial (those who, despite their low compliance were willing to participate and reported good tolerance) or 20 days after stating the study medication (right bars in each panel) when allocated to active treatment or placebo.

Figure 3 shows the overall treatment adherence and the incidence of moderate-to-severe side effects by the end of the run-in period (left) and day 20 after randomization (right). During the study, adherence decreased over time. The run-in demonstrated a sharp contrast in compliance (taking less than 80% of the study medication) between excluded and randomized (*P* <0.001). Although only 4.8% (17 of 351) participants in the run-in phase were considered noncompliant, this proportion increased to 12.1% by day 20 (36 of 297 randomized), with no difference between those assigned to placebo (14 of 126) or receiving trypanocidal therapy (22 of 171, *P* = 0.781). In regard to side effects, incidence was also clearly higher among excluded participants in comparison with the randomized, after the run-in (13 of 44 versus 38 of 307, *P* = 0.005); it changed to be numerically (but not statistically) lower by day 20 in the placebo group in comparison with those assigned to trypanocidal therapy (26 of 126 versus 51 of 171, *P* = 0.092).

The distribution of side effects reported during the run-in phase and on day 20 among those exposed to active treatment or placebo is shown in Table 2. When comparing excluded participants with those randomized (left part of the table), a higher frequency of any gastrointestinal symptoms (incidence 15.9% versus 4.6%, *P* = 0.018), nonspecific symptoms (13.6% versus 4.2%, *P* = 0.042) and musculoskeletal symptoms (9.1% versus 1.6%, *P* = 0.033) was observed. The new distribution of participants allowed for identifying side effects more attributable to each active drug on day 20 (right side of the table) such as nonspecific side effects (more often reported when taking nifurtimox [15.5%] than benznidazole [10.5%] or placebo [4.8%], *P* = 0.032) and skin reactions (12.8% when exposed to benznidazole, 6.0% when taking nifurtimox and 3.2% placebo, *P* = 0.022), but not other types of side effects.

**Table 2:** Moderate to severe side effects reported at the end of the run-in phase and onward by treatment allocated by day 20 after randomization

Symptoms, *n* (%)	End of Placebo Run-In Phase			Treatment Allocated at Day 20			
	Excluded (*n* = 44)	Randomized (*n* = 307)	*P*-Value	Nifurtimox (*n* = 84)	Benznidazole (*n* = 86)	Placebo (*n* = 126)	*P*-Value
Gastrointestinal							
Heartburn	3 (6.8)	3 (1.0)	0.056	4 (4.8)	4 (4.7)	2 (1.6)	0.340
Dyspepsia/flatulence	2 (4.6)	7 (2.3)	0.628	1 (1.2)	4 (4.7)	–	0.033
Nausea	2 (4.6)	2 (0.7)	0.156	–	1 (1.2)	2 (1.6)	0.524
Abdominal pain	3 (6.8)	3 (1.0)	0.056	3 (3.6)	1 (1.2)	1 (0.8)	0.280
Diarrhea	–	3 (1.0)	1	1 (1.2)	1 (1.2)	2 (1.6)	0.955
Anorexia	1 (2.3)	–	0.250	–	–	–	–
Constipation	1 (2.3)	1 (0.3)	0.470	1 (1.2)	1 (1.2)	1 (0.8)	0.948
Vomiting	1 (2.3)	–	0.250	–	–	2 (1.6)	0.257
Any	7 (15.9)	14 (4.6)	0.018	8 (9.5)	8 (9.3)	7 (5.6)	0.471
Nonspecific							
Asthenia	4 (9.1)	8 (2.6)	0.100	6 (7.1)	2 (2.3)	2 (1.6)	0.075
Choluria	1 (2.3)	1 (0.3)	0.470	4 (4.8)	1 (1.2)	1 (0.8)	0.108
Depression	1 (2.3)	2 (0.7)	0.663	5 (6.0)	5 (5.8)	3 (2.4)	0.347
Palpitations	2 (4.6)	1 (0.3)	0.084	3 (3.6)	–	–	0.022
Fever and chills	3 (6.8)	–	0.003	2 (2.4)	1 (1.2)	1 (0.8)	0.611
Dyspnea	1 (2.3)	–	0.250	5 (6.0)	1 (1.2)	1 (0.8)	0.037
Edema	1 (2.3)	–	0.250	–	1 (1.2)	–	0.294
Bleeding	–	2 (0.7)	1	–	–	–	–
Any	6 (13.6)	13 (4.2)	0.042	13 (15.5)	9 (10.5)	6 (4.8)	0.032
Nervous System							
Headache	1 (2.3)	9 (2.9)	>0.999	9 (10.7)	6 (7.0)	6 (4.8)	0.258
Dizziness	1 (2.3)	5 (1.6)	>0.999	2 (2.4)	2 (2.3)	–	0.222
Paresthesia	2 (4.6)	4 (1.3)	0.332	1 (1.2)	4 (4.7)	2 (1.6)	0.249
Others	–	–	–	3 (3.6)	4 (4.7)	2 (1.6)	0.419
Any	3 (6.8)	17 (4.5)	0.939	12 (14.3)	13 (15.1)	9 (7.1)	0.129
Musculoskeletal							
Myalgias	4 (9.1)	5 (1.6)	0.033	2 (2.4)	7 (8.1)	6 (4.8)	0.226
Cramps	3 (6.8)	–	0.003	1 (1.2)	–	2 (1.6)	0.517
Others	–	–	–	–	1 (1.2)	–	0.294
Any	4 (9.1)	5 (1.6)	0.033	2 (2.4)	8 (9.3)	8 (6.3)	0.166
Skin							
Pruritus	–	1 (0.3)	>0.999	5 (6.0)	9 (10.5)	3 (2.4)	0.045
Rash	–	–	–	3 (3.6)	9 (10.5)	3 (2.4)	0.024
Any	–	1 (0.3)	>0.999	5 (6.0)	11 (12.8)	4 (3.2)	0.022

The counts of reported side effects changed over some treatment transitions, as shown in Figure 2. In the group receiving initially placebo and then active treatment (OFF-ON, *n* = 171), the composite outcome appeared in the first treatment period in 49 participants and receded/resolved in 13 (*P* <0.001). In the second treatment period (days 60–120), in the group having an OFF-ON transition (*n* = 61, third from top to bottom, right side of the figure), side effects emerged/worsened in 15 participants and receded/resolved in 5 patients (*P* = 0.025). These outcomes contrasted with those of the group who received placebo in two consecutive periods (OFF-OFF) during the initial period (*n* = 126, bottom at the left), in which this ratio was 15:10 (*P* = 0.317) and in the second period (*n* = 50, bottom table at the right, ratio 9:6, *P* = 0.606). A similar picture was observed when active treatment was continued (ON-ON, *n* = 110, ratio 16:12, *P* = 0.450). Finally, in the group that discontinued active treatment (ON-OFF, *n* = 44), the observed relationship on day 20 was 6:7, suggesting that side effects remained 20 days after stopping active treatment (*P* = 0.781).

Regarding safety markers, some alterations were observed in the excluded participants in comparison with those randomized, although no significant differences were found between experimental groups. At the end of the run-in phase, the most common alteration was in hemoglobin levels (below 12 g/dL in Bogotá, of higher altitude, and 11 g/dL in Bucaramanga, 16.8%), followed by creatinine (6.9%) and ALT (6.3%). These alterations were more common in excluded participants than in those randomized to treatment (hemoglobin: 19.0% versus 16.5%; creatinine: 18.6% versus 5.2%; ALT: 16.3% versus 4.9%), reflecting this exclusion criterion of the run-in. During the experimental treatment, these alterations were similarly distributed between the placebo and trypanocidal therapy groups (hemoglobin: 11.0% versus 9.6%; creatinine: 5.8% versus 7.4%; ALT: 7.4% versus 6.2%).

## DISCUSSION

The run-in phase is a useful design tool that improves adherence to the protocol, allowing for an internally valid evaluation of the safety of the interventions.^32^^,^^35^ This tool is especially relevant in situations where the risk–benefit relationship is debatable, a circumstance that may negatively influence adherence, as can happen with the use of conventional trypanocidal agents. In our study, the run-in phase selected more adherent and tolerant participants before they received trypanocidal therapy, allowing a more accurate estimation and better discrimination of side effects. This advantage is reflected in the safety analysis conducted on day 20 of experimental treatment, where we observed significant differences in the report of side effects more likely attributable to trypanocidal therapy, such as nonspecific and cutaneous symptoms. In addition, the emergence of these side effects was consistent with the introduction of trypanocidal therapy (OFF-ON periods) at different times and in various subpopulations. These findings came at the cost of inducing some degree of nocebo effect and losing certain participants in the run-in period.

Our study appears to be the first including a run-in phase in its design to evaluate trypanocidal therapy. Other clinical trials have evaluated benznidazole at similar doses: 300 mg/day (MULTIBENZ,^36^ CHAGASAZOL,^37^ BENEFIT,^16^ STOP-CHAGAS,^12^ TRAENA)^15^^,^^38^ or 150 mg/day for 60 days (MULTIBENZ). When analyzing the sample included in the per-protocol (PP) analysis as a proxy for adherence, our study showed 90% adherence, exceeding all other reports: the 84% adherence seen in BENEFIT, 75% in TRAENA, both of which used doses of 300 mg/day; for the 150 mg/day benznidazole dose, the PP population was 81% in MULTIBENZ, whereas 87% of the participants in the EQUITY trial adhered, despite the longer treatment duration (120 days versus 60 days in MULTIBENZ), as shown in Supplemental Table 1. The higher adherence rates in our study PP analysis can be attributed to the selection of more committed individuals to follow the protocol. Currently, no data are available to compare the effects of a run-in phase in our study with other experimental studies of nifurtimox.

In our study, where safety assessment was crucial, we ensured that treatment remained indistinguishable for participants, study personnel, and outcome assessors. In addition, we included a placebo treatment as one of the experimental arms and completed the remaining treatment period of the participants assigned to trypanocidal therapy for 60 days through randomization. These features enhanced the validity of our study and allowed for the analysis of medication transitions with introduction or suppression periods (ON-OFF), facilitating inferences about the emergence or reduction of side effects.

However, these strengths are accompanied by several limitations. First, using a run-in phase inherently selects participants, which may limit the generalizability of the results. This approach could affect understanding of the safety profile in specific subgroups, such as those with comorbidities or those receiving other medications that might cause interactions or intolerance.^39^ Second, the safety assessment involved recording relatively infrequent events, which required sufficient statistical power for their identification. Our study, like most clinical trials, estimated its sample size based on efficacy outcomes. As a result, some potentially important safety events may not have been identified due to limited statistical power or follow-up duration. In addition, we compared the occurrence of these events at a single, arbitrary time point (20 days after starting each 60-day treatment period). This single observation could have underestimated the report of some side effects appearing earlier or later (e.g., transient skin reactions or neurological symptoms). Also, the analysis of before-and-after changes in subgroups and periods depended on the timing of evaluations. In the case of active drug suppression (ON-OFF situations), a 20-day “washout” period may not have been long enough to observe a potential reduction in symptoms. Furthermore, this evaluation did not determine the extent of these phenomena nor establish causality. In our study, the effects of the run-in phase were evaluated 10 days after initiation, whereas in the experimental phase assessments were conducted 20 days after treatment initiation. These differences in timing may limit comparability between the two phases.

Finally, this trial did not include detailed clinical staging for participants but rather took a pragmatic approach for inclusion. Eligibility required a positive *T. cruzi* serology and not having a symptomatic cardiomyopathy, based on clinical judgment alone. However, having small proportions of prescribed drugs for heart failure and just one participant with BNP levels over 100 pg/mL gives confidence that very few participants were beyond the stage A of heart failure.^40^

From a practical perspective, the EQUITY study provides valuable findings regarding adverse effects that clinicians can expect from trypanocidal therapy. Adverse events—aside from nonspecific symptoms and skin reactions—did not occur at significantly higher rates or with sufficient frequency to raise significant concerns, at least in the short term. These results suggest that such adverse effects should not significantly impact adherence, at least during the first 20 days of treatment. A recent trial suggested that shorter treatments with benznidazole are better tolerated while maintaining antiparasitic efficacy.^18^ Recognizing an acceptable safety profile can enhance communication about the risk–benefit balance, support the initiation of symptomatic management when needed, and encourage greater adherence.

From a research perspective, our findings highlight the potential for a nocebo effect when obtaining informed consent and during follow-up data collection.^41^ In such situations, negative reactions—even to a substance without clinical effect—may be induced in participants. The nocebo effect may lead to an overestimation of adverse events, influence adherence, and ultimately affect the efficiency of clinical trials.^42^ This effect may be exacerbated if participants receive safety information framed negatively (e.g., “1 in 10 people do not finish the medication”) or if follow-up visits focus excessively on specific symptoms instead of adopting an open, more neutral, or simply expectant approach to discussing adverse reactions.

In the evaluation of health interventions, there is often a trade-off between pragmatism and inclusiveness regarding study efficiency. To balance these aspects, future studies should consider emotional factors, participants’ values and preferences regarding participation, the nature of their disease, and the experimental treatment.^41^ Furthermore, how information is delivered is crucial, because acknowledging the nocebo effect may mitigate its impact. One approach is “contextualized informed consent,” in which participants are educated about the nocebo effect and given the option to decide whether they wish to be informed about minor adverse effects. This strategy could help optimize communication, reduce bias from expectations, and improve the evaluation of the safety of therapeutic interventions.

## CONCLUSION

In this study, the run-in phase with placebo facilitated the selection of participants with higher adherence rates. In addition, it helped identify symptoms associated with treatment administration and their temporal relationship with its initiation. However, these advantages came at the cost of potentially inducing a nocebo effect in excluded run-in participants, which limits the generalizability of the safety results. When evaluating trypanocidal therapy or therapies in similar contexts, it is crucial to optimize methodologies that provide a more comprehensive assessment of the risks and benefits of interventions.

## Supplemental Materials

10.4269/ajtmh.25-0198 Supplemental Materials
