# Supplementary material for: Value of the Run-In Period to Evaluate the Safety of Conventional Trypanocidal Treatment: A Subanalysis of a Colombian Randomized Clinical Trial
Source: Am J Trop Med Hyg. 2026 Apr 21;114(6):1148–56. doi: 10.4269/ajtmh.25-0198 (PMC13235602; doi:10.4269/ajtmh.25-0198)
Supplement: Supplemental Materials [file tpmd250198.SD1.pdf]

Supplemental Table 1
Summary of randomized clinical trials in chagas disease:
Adherence to different benznidazole dosing

Randomized Controlled Trial	**Dosing**	**Intention-to-Treat Population**		**Per-Protocol Population**	
	**Standard dose**	**Placebo *n* (%)**	**Benznidazole *n* (%)**	**Placebo *n* (%)**	**Benznidazole *n* (%)**
MULTIBENZ [18]	benznidazole 300mg x 60d	N/A	78 (100)	N/A	53 (68)
CHAGAZASOL [37]	benznidazole 300mg x 60d	N/A	26 (100)	N/A	17 (65)
BENEFIT [16]	benznidazole 300mg x 40-80d	1423 (100)	1431(100)	1338 (94)	1202 (84)
STOP-CHAGAS [12]	benznidazole 400 mg x 60d	30 (100)	30 (100)	27 (90)	16 (53)
TRAENA [15,38]	benznidazole 300mg x60d	357 (100)	352 (100)	350 (98)	263 (75)
EQUITY	benznidazole 300mg x 60d	61 (100)	60 (100)	55 (90)	54 (90)
	**Alternative doses**				
MULTIBENZ	benznidazole 150mg x 60d	N/A	77 (100)	N/A	62 (81)
EQUITY	benznidazole 150 mg x120d	61 (100)	62 (100)	55 (90)	54 (87)
